# Effect of artificial shading on the tannin accumulation and aromatic composition of the Grillo cultivar (*Vitis vinifera* L*.*)

**DOI:** 10.1186/1471-2229-13-175

**Published:** 2013-11-06

**Authors:** Pietro Scafidi, Antonino Pisciotta, Davide Patti, Pasquale Tamborra, Rosario Di Lorenzo, Maria Gabriella Barbagallo

**Affiliations:** 1 Department of Agricultural and Forest Sciences, Università degli Studi di Palermo, Viale delle Scienze 11 ed. H, Palermo 90128, Italy; 2 CRA-UTV, Via Vittorio Veneto 26, Barletta, BA 70051, Italy

**Keywords:** Sunlight, Temperature, Catechins, Proanthocyanidins, Flavour compounds

## Abstract

**Background:**

White wine quality, especially in warm climates, is affected by sunlight and heat stress. These factors increase the probability that ambering processes will occur and reduce the potential flavour compounds. This study aimed to investigate the effect of sunlight reduction on the accumulation of polyphenolic and aromatic compounds.

**Results:**

This study was conducted in a commercial vineyard containing *V. vinifera* L. cv Grillo. Opaque polypropylene boxes (100% shading) and high-density polyethylene (HDPE) net bags (50% shading) were applied at fruit set. The effect of the shaded treatments was compared to the exposed fruit treatment. The shaded treatments resulted in heavier berries and lower must sugar contents than the exposed treatments. Proanthocyanidins and total polyphenol levels were similar in the exposed and bagged grapes; however, the levels were always lower in the boxed fruit. At harvest, the highest aroma level was measured in the boxed fruits.

**Conclusions:**

The boxed fruit had less sugar, fewer proanthocyanidins and more flavours than the exposed grapes.

The reduction in flavanols reactive to p-dimethylamino-cinnamaldehyde as (+)-catechin equivalents and total skin proanthocyanidins is an important result for the white winemaking process. In addition, the higher level of aromatic compounds in shaded grapes at harvest is an important contribution to the development of different wine styles.

## Background

The effects of sun exposure on grape composition are vast and complex [[1]. The radiation and heat from sunlight can influence metabolic reaction rates and cause stress, either by dehydration or by a direct increase in temperature [[2]. It is generally accepted that shade results in significant alterations in grape composition and reduces wine quality [[3].

### Effects of light on juice composition and berry weight

Several studies [[4-6] have shown that grapes grown in low-light conditions have lower soluble solid contents [[7,8], lower pH levels and higher titratable acidity [[8] (in particular, higher concentrations of malic acid) than fruit cultivated in high-light conditions. No significant differences in the total soluble solids in the juice or in the berry weight was found, comparing the compositions of berries sampled from bunches that were either fully exposed or completely artificially shaded [[9].

### Effects of light on the content and composition of anthocyanins and flavonols

The levels of anthocyanins were markedly reduced by shading [[3,10]. Previous work has shown that the reduction in the anthocyanin concentrations in fully exposed clusters of “Norton” grapes was likely a result of the berry temperatures, which were higher than the ambient temperature [[8]. In particular, fruit shading decreased the 3′-hydroxylated anthocyanin concentration and increased the 3′,5′-hydroxylated anthocyanin concentration [[11]. In contrast with previous results, the level of quercetin-3-glucoside per berry was significantly higher in berries collected from exposed bunches [[9,12-14].

### Effects of light on the proanthocyanidin content

The concentrations of proanthocyanidins in the berry skin were higher in the exposed clusters [[2,10,15], but it was similarly reported that the concentrations of proanthocyanidins in the berry skin were similar in boxed and sun-exposed bunches, although the compositions were different [[14]. A higher content of proanthocyanidins may be beneficial in black grape varieties because they produce red wines with good structure; however, in white grape varieties, high levels of proanthocyanidins may be problematic. High levels of polyphenols increase the likelihood of the formation of brown polymers during vinification [[16].

### Effects of light on the aromatic composition

Sun exposure strongly influences the aromatic composition of grapes. Excessive exposure to sunlight and high berry temperatures reduces the content of methoxpyrazines [[17,18]. However, the glycosidically bound monoterpenes and polyols (potentially volatile terpenes) in Gewürztraminer grapes [[19] and the norisoprenoid concentrations in Weisser Riesling and Chenin Blanc grapes [[20] were maintained at the highest levels in exposed berries during ripening and were considerably higher than in the partially and completely shaded fruit at harvest. Wines produced from shaded fruit contained lower levels of glycosides, β-damascenone and 1,1,6-trimethyl-1,2-dihydronaphthalene (TDN) [[21]. In Shiraz grapes, quantitative decreases in the levels of glycoconjugates were observed in artificially shaded bunches, particularly for C13-norisoprenoidic glycosides [[22]. However, in a comparison between shaded, partially shaded and fully exposed treatments, the final concentrations of β-damascenone and β-ionone in Shiraz grapes and wine were not significantly affected by the sun exposure levels [[23]. The concentrations of free and bound terpenol in shaded Chilean Muscat berries were so low that the characteristic muscat aroma was lost, which resulted in poor-quality musts and wines [[24]. In warm environmental conditions, heat and sunlight stress can reduce the aromatic content of grapes, as confirmed in Sicily by Costanza et al. [[25], who showed there was negative effect of early cluster zone defoliation on the flavour compounds of the Grillo cultivar and on glycosylated aromatic composition.

### Study aim and objectives

The purpose of this study was to investigate the impact of shading on the composition of Grillo, an indigenous white Mediterranean variety cultivated in Western Sicily on more than 4,000 ha [[26]. While Grillo is generally used to produce “Marsala” wine, it has recently been used to produce new styles of table wines [[27].

In previous studies, the effect of shading utilising opaque polypropylene boxes or net bags was investigated on black varieties [[11,14,28-31]. Based on results obtained it is anticipated that shading will alter the composition of Grillo grapes by lower polyphenol concentration and increasing volatile aromatics and the glycosylated precursors.

## Methods

This study was performed in 2009 in a non-irrigated commercial vineyard of cv. Grillo *(V. vinifera* L), located in the “Alcamo DOC” area (Sicily 37° 54′ 14.94′ N – 13° 06′ 08.53′ E). The vines were grafted onto 140 Ruggeri rootstock, trained on vertical shoot positions and pruned leaving one cane of eight buds per vine. Rows oriented in a north–south direction were spaced 2.4 m apart, whereas in-row vine spacing was 1 m. To study the effects of different light conditions during ripening, three light environments were evaluated, completely shaded (boxed), partially shaded (net-bagged) and fully exposed.

The boxes were designed [[14] to maintain airflow, exclude light and minimise changes in temperature and humidity. They were made from a polypropylene sheet (0.6 mm), and the boxes were white on the outside and black inside. The box dimensions were 25 × 20 × 10 cm. The boxes (100% shading) and high density polyethylene (HDPE) net bags (50% shading) were applied to grape bunches located on the east side of the canopy from fruit set to harvest. Fifty vines were subjected to the experiment, and one box and one net-bag were placed on each vine. All of the vines were defoliated in the bunch zone immediately prior to the application of the covers to prevent the leaves from shading the uncovered bunches. Shaded samples were compared with the exposed east-side samples taken from the same vines. Three field replicates of three vines each were used for each sampling date. From the end of veraison (August 5^th^) to harvest (September 9^th^), three bunches per treatment from each field replicate were sampled approximately every 10–15 days (four sampling dates in total). For each treatment and replicate, 25 berries were randomly collected. Each 25-berry sample was first weighed, next, the skins were separated from the flesh, and the proanthocyanidin index [[32] and the reactivity of flavanols to p-dimethylamino-cinnamaldehyde (p-DAC assay) as (+)-catechin equivalents [[33] were determined using a UV–vis spectrophotometer (Varian Cary 50 Bio UV-Visible Spectrophotometer, McKinley Scientific, Sparta, New Jersey, USA).

The flesh of each 25-berry sample, when separated from the skins, was crushed, centrifuged and juice total soluble solids (°Brix) and titratable acidity measured. Titratable acidity was expressed as g/L of tartaric acid [[34].

At harvest, 100 berries from each of the three field replicates per treatment were collected, to determine the volatile and bound compounds by enzymatic hydrolysis using GC-MS [[35].

In all treatments, from fruit set (June 16^th^) to harvest (September 9^th^), the temperature inside the bunches was recorded every 60 minutes using needle sensors (Ø 0.5 cm). From July 22^nd^ to August 2^nd^, the hourly solar radiation (watt/m^2^) in the open air and in the bunch zone was recorded. All sensors were connected to a WatchDog data-logger (Spectrum Technologies, Inc.).

### Statistical analysis

Means and standard errors were reported. Analysis of variance (ANOVA) and Tukey’s HSD test was used at a 5% level of significance (α = 0.05). Lowercase letters indicate statistically significant differences at a 5% level of significance. All statistical analyses were performed using SYSTAT 10.

### Extraction and determination of tannins

#### Preparation of skin extract

The skins of the 25-berry samples were separated from the pulp and placed in a flask containing 25 mL of tartaric buffer (pH 3.2) (produced by adding the following chemicals in the following order: 500 mL of distilled water, 5 g of tartaric acid, 22 mL of 1 N NaOH, 2 g of sodium metabisulphite and 120 mL ethanol 95%). The volume of buffer was adjusted to 1 L by the addition of distilled water. Skins were placed in the buffer for four hours at room temperature prior to homogenisation and centrifugation. The supernatant was collected in a 100 mL volumetric flask, the residue was washed again with tartaric buffer (pH 3.2) added to the volumetric flask and the volume was raised to 100 mL with tartaric buffer (pH 3.2).

#### Proanthocyanidins index

The skin extract (0.2 mL) was placed in a 50-mL distillation tube in cold water. Ethanol 96% (12.3 mL) and HCl containing 300 mg/L of FeSO_4•_7H_2_O (12.5 mL) were added to the tube, and the absorbance spectrum from 360 to 700 nm was recorded (E_0_). Next, the tube containing the solution was placed in boiling water. After 50 minutes, the absorbance spectrum from 360 to 700 nm was recorded again (E_1_).

The results were calculated using the following equation: (E_1_-E_0_) × 1162.5 × (1/0.2_ml_) × (100_ml_/1000)/25 [[32].

#### Flavanols p-dimethylamino-cinnamaldehyde reactive index

p-Dimethylamino-cinnamaldehyde (100 mg) was dissolved into 70 mL of methanol and added to 25 mL of concentrated HCl. The volume was then adjusted to 100 mL with methanol. The skin extract was diluted 10 times with distilled water. Diluted skin extract (1 mL) was placed in a tube containing 5 mL of p-dimethylamino-cinnamaldehyde solution. After 10 minutes, the absorbance at 640 nm was read (E_1_). The instrument was zeroed, and the absorbance of a solution of 5 mLp-dimethylamino-cinnamaldehyde reagent with the addition of 1 mL of distilled water (E_0_) was recorded, followed by the measurement of the absorbance of 5 mL of distilled water added to 1 mL of diluted skin extract (E_00_).

The results were calculated using the following equation: Flavanols p − dimethylamino − cinnamaldehyde reactive index (mg/100 berries) = 38.88 × [(E_1_ − E_0_ − E_00_)–0.34] × (1/0. 1_mL_) × (100_mL_/1000) × 4 [[33].

### Preparation of aroma extract samples

At harvest, 100 berries per replicate (three replicates) were randomly sampled from each treatment and weighed. The seeds were removed, and the skins were placed in a flask containing methanol, while the pulp was collected in a flask containing sodium metabisulphite. The skins remained in methanol for one hour to inactivate the glucosidase enzymes [36]. The two phases were then combined and homogenised using an immersion blender. The homogenate was centrifuged, and the supernatant was collected in a volumetric flask. The solid phase was three times washed and then resuspended in 40 mL of pH 3.2 buffer (produced by adding the following chemicals in the following order: 500 mL of distilled water, 5 g of tartaric acid, 22 mL of 1 N NaOH and 2 g of sodium metabisulphite). The suspension was centrifuged and the supernatant was added to the volumetric flask. The volume of supernatant was raised to 400 mL with pH 3.2 buffer. Pectolytic enzyme (Enartis ZYM 1000 S, Esseco s.r.l, Italy) (200 μL) devoid of secondary activity was added to the volumetric flask and incubated for at least one hour, and the extract was filtered.

### Aroma extraction and determination

The filtered extract was passed through a 5 g Isolute C18 cartridge (International Sorbent Technology, UK), which was activated by adding 20 mL of methanol and 50 mL of double-distilled water. Next, 30 mL of dichloromethane was passed through the cartridge to elute the free volatile compounds. The bound fraction were eluted using 30 mL of methanol [35].

### Analysis of free volatile compounds

The dichloromethane extract was frozen at −16°C to eliminate the water component, and the remaining dichloromethane phase was poured into a 100-mL volumetric flask. It was then dried by the addition of anhydrous sodium sulphate and concentrated by distillation. Once concentrated to a volume of 0.2 mL, this fraction was analysed using GC-MS [37].

### Analysis of glycosylated compounds

The investigation was carried out through GC-MS determination of volatile compounds obtained by the enzymatic hydrolysis of glycosides present in grapes [[35].

The glycosylated aroma extracts (methanol extract) were evaporated under vacuum at 40°C, and the residue was recovered using 5 mL citrate-phosphate buffer at pH 5. In each aroma extract, 0.4 mL of a high-glycosidic-activity enzyme (Citolase FL, Genencor Inc., San Francisco, CA) was added, and the solution was incubated at 40°C for 24 hours. An aliquot (1 mL) of internal standard (10 mg L^-1^ of 1-heptanol in 40% ethanol) was added after incubation, and this mixture was then passed through a 1 g Isolute C18 cartridge (International Sorbent Technology, UK), which was activated prior to the experiment using 5 mL of methanol and 10 mL of double-distilled water.

The column was washed with 10 mL of water, and the aglycones produced by enzymatic hydrolysis of the glycosylated forms were eluted using 6 mL of dichloromethane. The dichloromethane extract was dried by the addition of anhydrous sodium sulphate, concentrated to 0.2 mL under a stream of nitrogen and analysed using GC-MS. Two microliters of the concentrate were injected into a HP-FFAP fused silica open tubular column (30 m × 0.25 mm × 0.25 μm) (Agilent, Palo Alto, CA, USA) with a splitless system for one min [35].

### GC-MS conditions

GC-MS analysis was performed using a 5890 gas chromatograph interfaced with a 5972 mass selective detector (Agilent, Palo Alto, CA, USA). The identification of compounds was performed using a NIST 05 library (using a percent matching higher than 95% as the threshold value for acceptance) and comparing the linear retention index and the electron impact (EI) mass spectra with data from reference compounds. The concentration was calculated as 1-heptanol (internal standard). The determination of different compounds was calculated as follows:

*μ*g/L : Sx × Cs × Ss^− 1^

*μ*g/100 berries :  *μ*g/L × V × 1000–1 [[35].

(Sx is the area of the compound, Ss and Cs are the area and the concentration of the internal standard, respectively, and V is the volume (mL) of the extract).

## Results

Light was excluded within the box, while the light reaching the net-bagged bunches was reduced by more than 70% from 7:00 am to 12:00 pm. The shading was the result of both the net-bag (50% shading) and the shade produced by the leaves and lateral shoots, located above the grape bunches that grew during the vegetative season. For the same reason, the grape bunches in the exposed treatment (defoliated in the bunch zone) were shaded by approximately 50% during the mornings. After midday, the shading increased to approximately 90% in the net-bagged treatment and to approximately 80% in the exposed bunches. This difference was due to the row orientation (N-S) that naturally increased the shade on the east side of the row (Table 1). Taking into consideration the number of hours in which the temperatures were above 35°C, the boxed bunches spent only 231 hours above 35°C from June 16^th^ to September 9^th^, while the exposed bunches spent 378 hours (a 39% increase) above that temperature. The lack of ventilation inside the net-bags resulted in a higher average of temperatures over 35°C but in a lower number of hours over 35°C (Table 2).

**Table 1:** **Reduction (%) in solar radiation during the day from July 22nd to August 2**^**nd**^

**Time**	**07.00**	**08.00**	**09.00**	**10.00**	**11.00**	**12.00**	**13.00**	**14.00**	**15.00**	**16.00**	**17.00**	**18.00**	**19.00**	**20.00**
**Treatment**	**%**													
Exposed	11.1	48.9	48.8	48.5	50.8	47.9	80.6	80.8	86.4	85.4	79.5	71.7	64.8	91.4
Net-bagged	72.6	75.2	71.5	69.8	79.9	74.4	90.9	81.5	92.9	92.9	91.9	89.2	88.8	100
Boxed	100	100	100	100	100	100	100	100	100	100	100	100	100	100

**Table 2:** **Sum of the number of hours in which the bunch temperatures were greater than 35°C and monthly averages of temperatures above 35°C from June 16**^**th **^**to September 9**^**th**^

**Month**	**June**		**July**		**August**		**September**	
**Treatment**	**Nr. Hours**	**Average T > 35°C**	**Nr. Hours**	**Average T > 35°C**	**Nr. Hours**	**Average T > 35°C**	**Nr. Hours**	**Average T > 35°C**
Exposed	18	37.78	172	38.73	163	38.85	25	38.04
Net-bagged	20	38.20	126	39.77	146	40.73	32	41.88
Boxed	11	36.18	107	37.23	101	36.46	12	35.58

**Figure 1:**
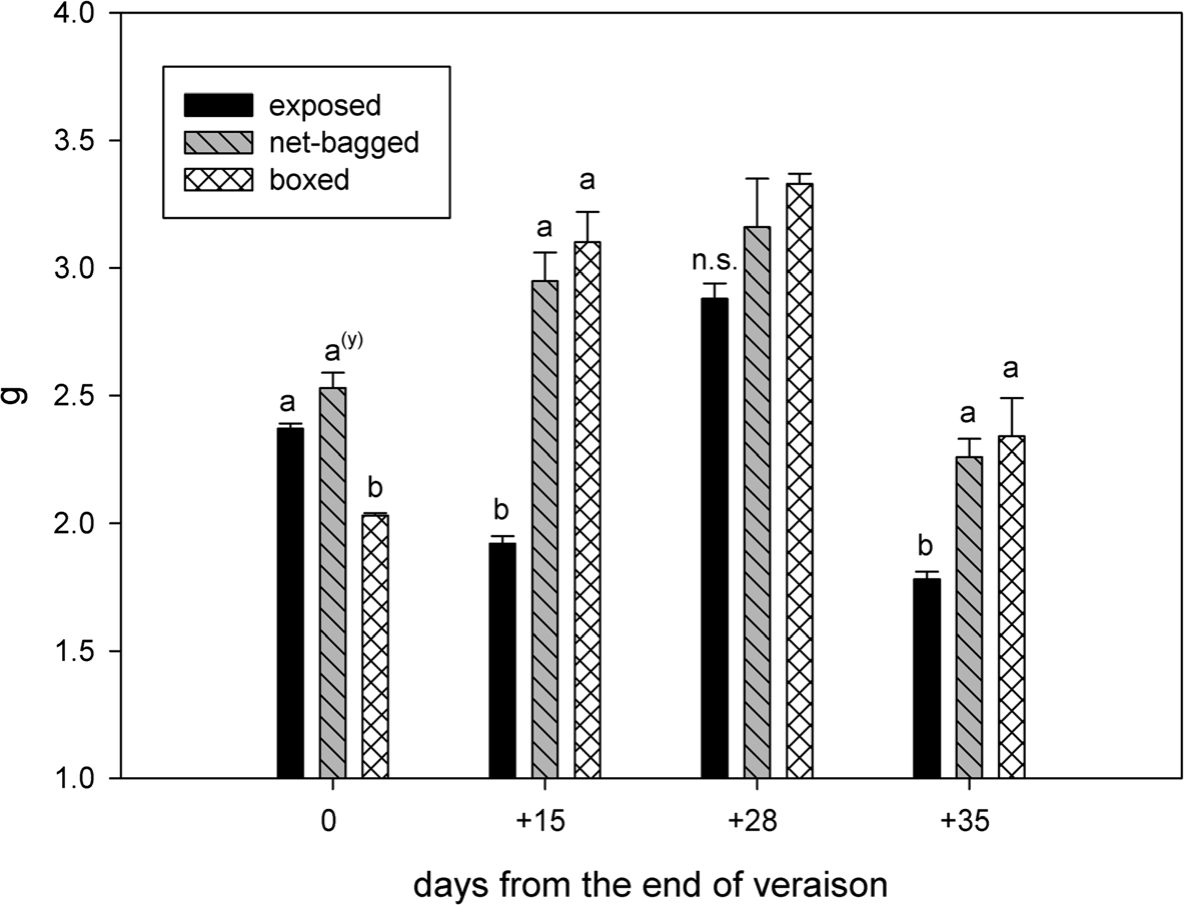
**Berry weight (g) from the end of veraison to harvest.** Values represent means ± SE (n = 3). ^(y)^ Lowercase letters indicate statistically significant differences at a 5% level of significance. (Tukey’s HSD test), n.s. = not significant.

**Figure 2:**
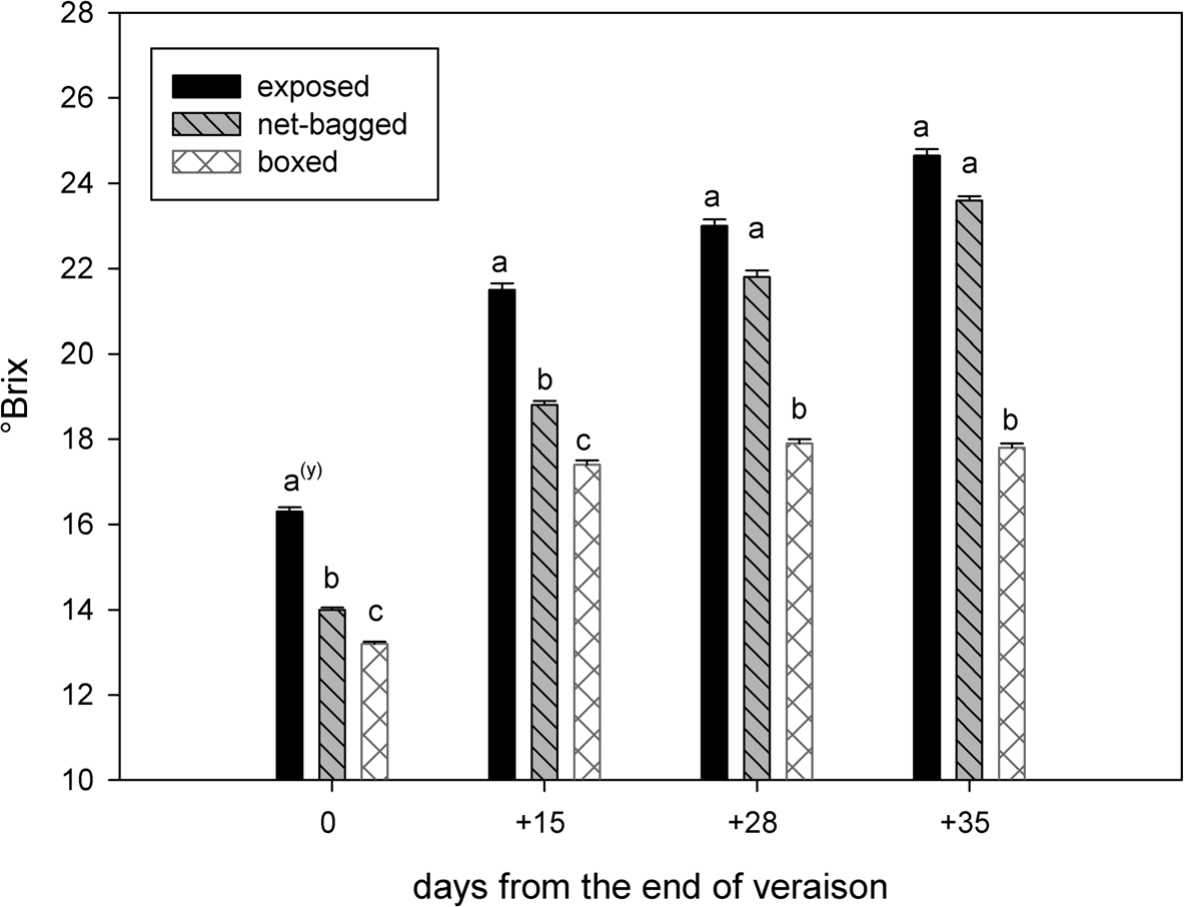
**Total soluble solids (°Brix) from the end of veraison to harvest.** Values represent means ± SE (n = 3). ^(y)^ Lowercase letters indicate statistically significant differences at a 5% level of significance. (Tukey’s HSD test).

The berries boxed at fruit-set were the lightest (2.03 g) at veraison (August 5^th^). After this stage, they grew quickly, and at harvest (September 9^th^) they were the heaviest (2.34 g). After August 5^th^ the exposed berries always weighed the least. Few differences in weight were noted between the boxed and net-bagged berries. At harvest, reductions in berry weight were recorded for all treatments; however, the largest reduction occurred in the exposed berries (a decrease of 38.2%) (Figure 1). The solid soluble content was always lower in boxed berries than in other treatments. At the end of veraison and 15 days later, the net-bagged berries contained fewer sugars than exposed berries (Figure 2). However, 28 and 35 days after the end of veraison, similar values of total soluble solids were observed in exposed and net-bagged berries.

**Figure 3:**
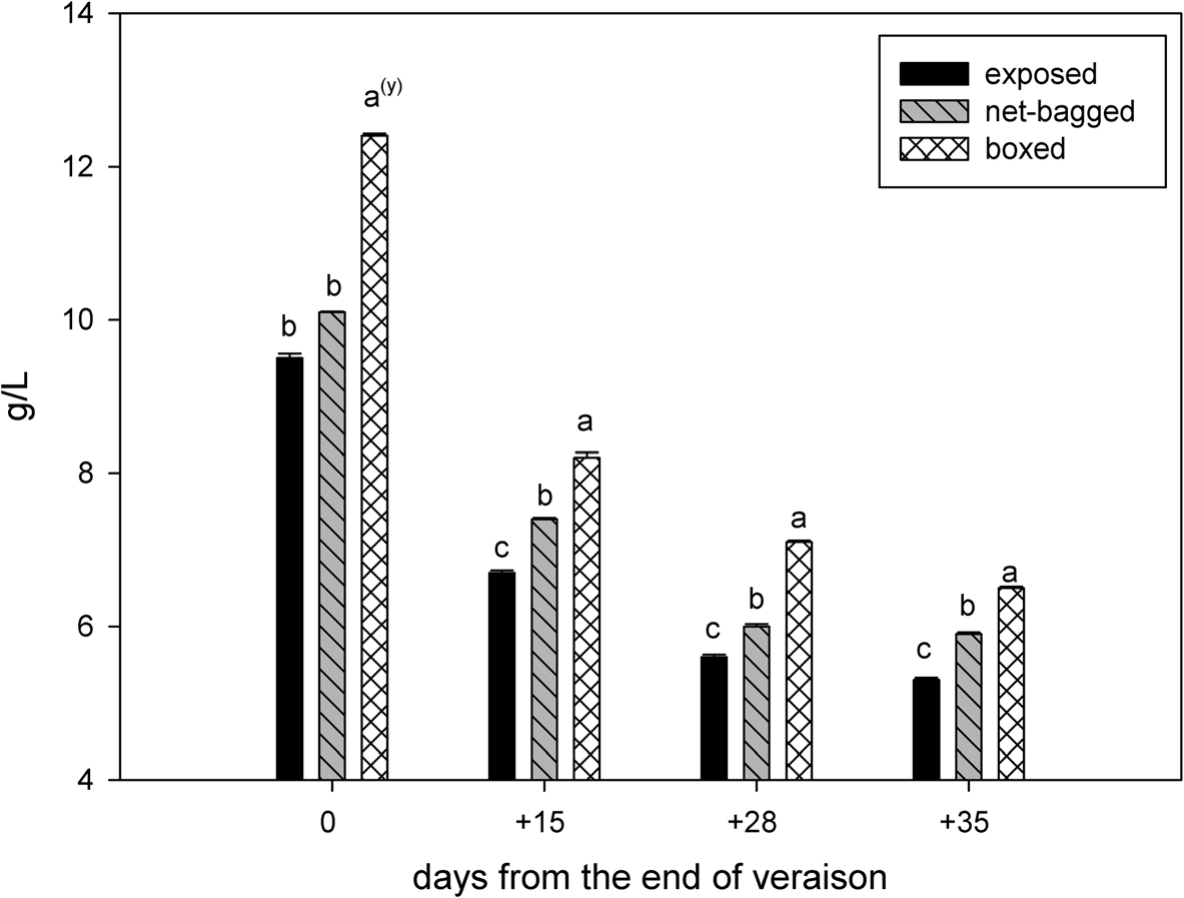
**Titratable acidity (g/L tartaric acid) from the end of veraison to harvest.** Values represent means ± SE (n = 3). ^(y)^ Lowercase letters indicate statistically significant differences at a 5% level of significance. (Tukey’s HSD test).

**Figure 4:**
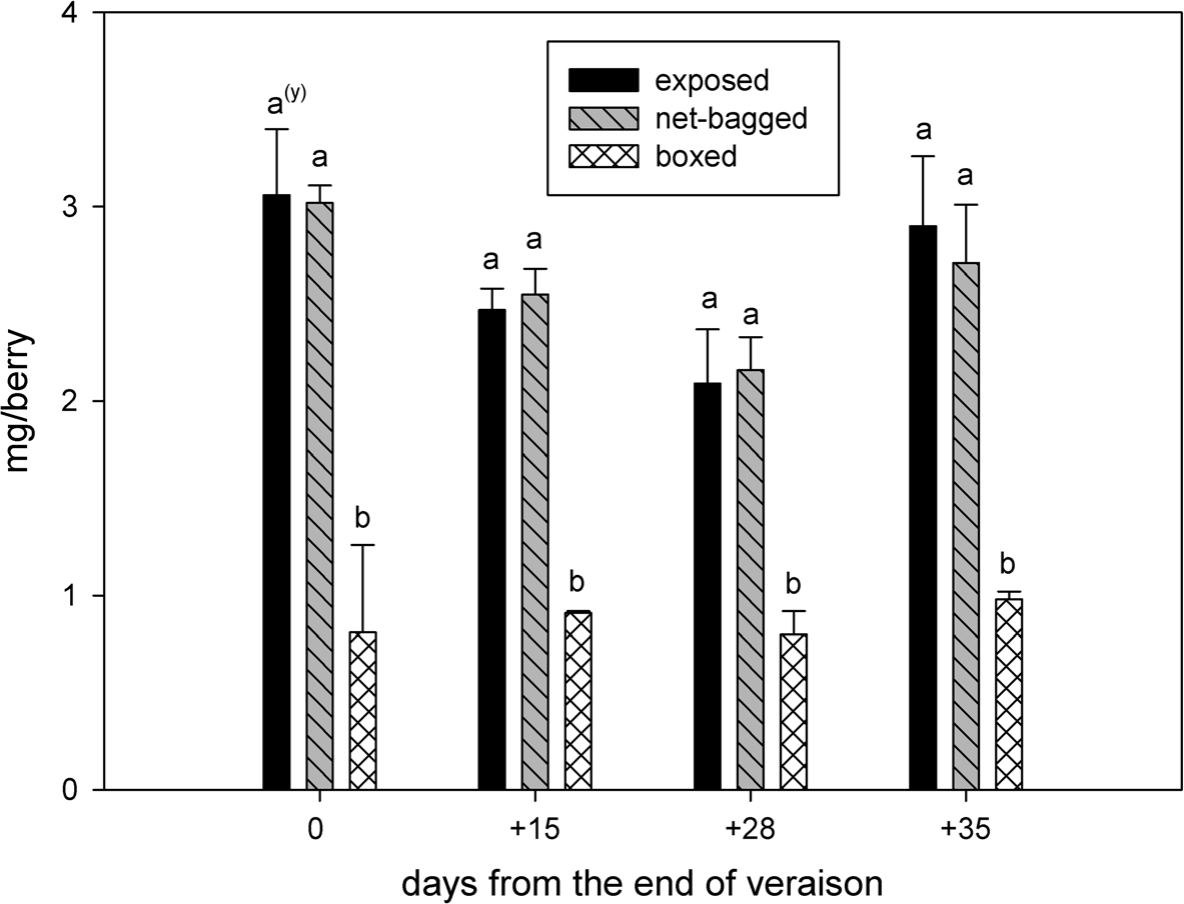
**Total skin proanthocyanidin (mg/berry) from the end of veraison to harvest.** Values represent means ± SE (n = 3). ^(y)^ Lowercase letters indicate statistically significant differences at a 5% level of significance.

**Figure 5:**
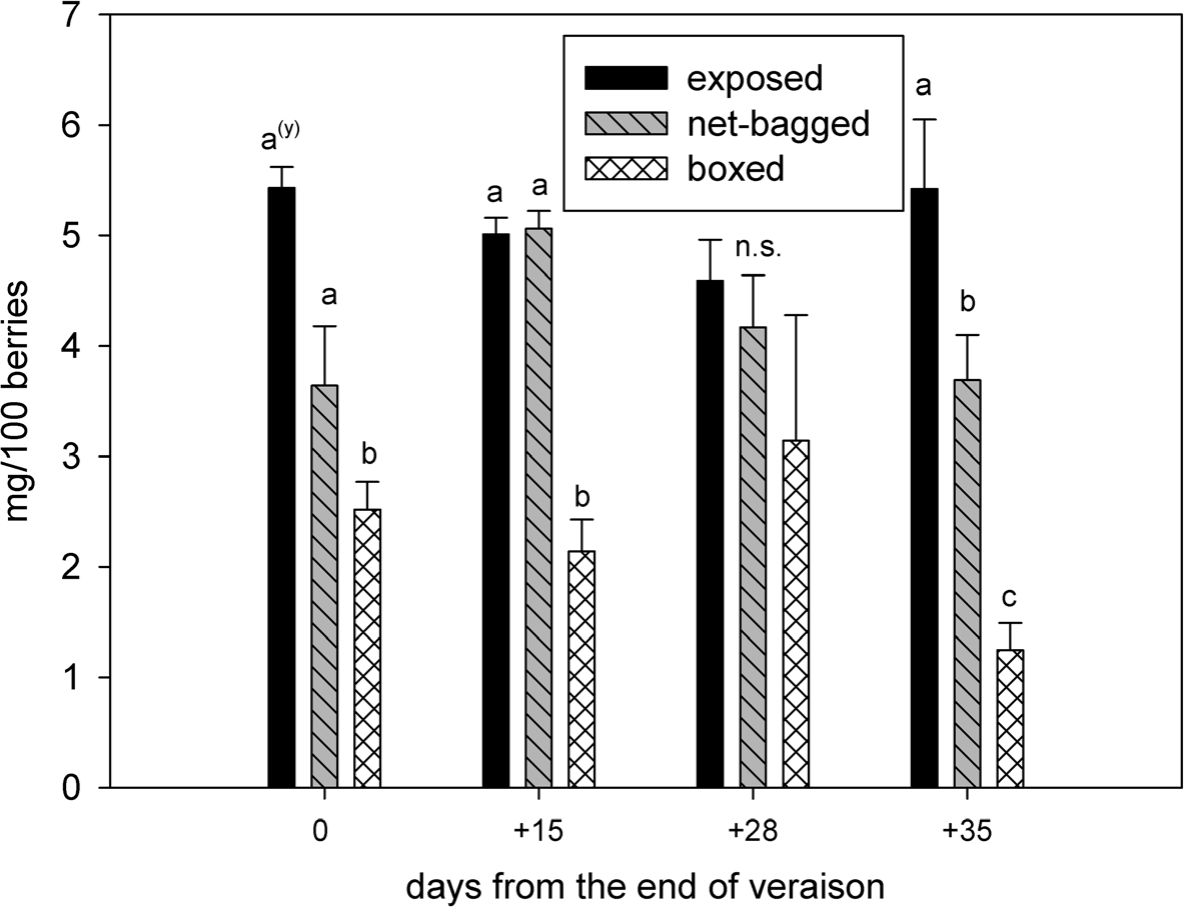
**p-DACA-reactive skin flavanols (mg/100 berries) from the end of veraison to harvest.** Values represent means ± SE (n = 3). ^(y)^ Lowercase letters indicate statistically significant differences at a 5% level of significance. (Tukey’s HSD test), n.s. = not significant.

The boxed berries had the highest values of titratable acidity during the entire ripening season, followed by net-bagged berries, and the lowest values were observed in the exposed berries (Figure 3). The proanthocyanidin content was similar in exposed and net-bagged grapes, although it was significantly lower in boxed berries (Figure 4). The p-dimethylamino-cinnamaldehyde-reactive flavanols were also lower in berries from boxed bunches (Figure 5).

The free volatile compounds were mainly aldehydes and six carbon (C6) alcohols derived from the enzymatic oxidation of unsaturated fatty acids. Free terpenes were absent, with the exception of traces of linalool. The benzenoids were also only detected in low quantities, as were traces of benzoic acid and vanillin. In addition, the lowest amounts of benzyl alcohol and 2-phenylethanol and the highest amounts of *trans*-2-hexenal were found in the net-bagged grapes (Table 3).

**Table 3:** Non-glycosylated aromatic composition of grapes at harvest

**Treatment**	**Exposed**			**Net-bagged**			**Boxed**		
	**μg/100 berries**								
	**mean**	**± SE**		**mean**	**± SE**		**mean**	**± SE**	
Hexenal	1.0	0.01	b	2.5	0.03	a	3.3	0.28	a
3-pentene-2-	5.2	0.15		5.6	0.04		4.2	0.06	n.s.
*Trans* 2-hexenal	11.2	0.11	c	33.8	2.22	a	20.7	1.82	b
1-hexanol	32.8	1.57	b	29.1	2.64	b	40.7	2.65	a
*Trans* 2-hexenol	56.0	4.69		46.6	3.50		55.1	4.23	n.s.
*Cis* 3-hexenol	9.4	0.23	b	11.2	0.33	a	12.0	0.42	a
*Cis* 2-hexenol	0.7	0.01		0.7	0.01				n.s.
Linalool	0.1	0.01							
Benzyl alcohol	11.8	0.31		7.4	0.16		12.5	0.68	n.s.
2-phenylethanol	23.7	0.07	a	12.5	0.16	b	28.3	2.33	a
Benzoic acid	1.5	0.05	b	3.3	0.05	a	2.1	0.11	b
Vanillin	1.4	0.06	b				3.7	0.23	a
Total	155.1			153.0	3.44		182.7		

Values represent means ± SE (n = 3).

Lowercase letters indicate statistically significant differences at a 5% level of significance (Tukey’s HSD test); n.s. = not significant.

In our experiment, the concentrations of non-glycosylated aromatic components were similar in exposed and net-bagged grapes, as found in Muscat of Alexandria grapes [[24], while the highest levels were measured in the boxed fruits (17.8% and 19.4% higher than the exposed and net-bagged grapes, respectively) (Table 3).

The same effects of shading on free fractions were observed in the bound fraction (Tables 4 and 5). The lowest concentrations of glycosylated aromatic compounds were found in exposed grapes. Six carbon (C6) alcohols were only detected in low quantities; the highest levels of these compounds were present in the boxed grapes (41% more than in the exposed treatments) (Table 5). Hexanol and *cis*-hexenol, associated with green aromas [[38] and related to the degree of grape ripening [[39], were the most highly represented compounds (Table 6).

**Table 4:** Glycosylated aromatic composition of grapes at harvest in μg/100 berries

	**Exposed**				**Net-bagged**				**Boxed**			
	**μg/100 berries**			**%**	**μg/100 berries**			**%**	**μg/100 berries**			**%**
**Treatment**	**mean**	**± SE**			**mean**	**± SE**			**mean**	**± SE**		
C6-alcohols	42.8	5.5	b	7.4	46.5	2.1	b	7.0	60.4	3.0	a	4.9
Benzenoids	417.6	63.4	b	72.0	523.5	18.0	b	78.9	1008.4	66.7	a	81.6
Terpenes	75.7	11.1		13.1	61.2	2.8		9.2	108.6	7.7	n.s.	8.8
C_13_-norisoprenoids	43.9	2.7		7.6	32.4	1.2		4.9	58.7	2.3	n.s.	4.7
Total	580.0				663.6				1236.2			

Values represent means ± SE (n = 3).

Partitioning among C6 alcohols, benzenoids, terpenes and C13-norisoprenoids is expressed as percentages (%).

Lowercase letters indicate statistically significant differences at a 5% level of significance.

(Tukey’s HSD test); n.s. = not significant.

**Table 5:** Differences (%) in flavours of the net-bagged and boxed treatments compared to the exposed treatment

**Treatment**	**Net-bagged**	**Boxed**
	**%**	
C6-alcohols	8.76	41.12
Benzenoids	25.24	141.47
Terpenes	−19.21	43.46
C13-norisoprenoids	−26.31	33.71
Total	14.32	113.13

**Table 6:** Glycosylated aromatic composition of grapes (μg/100 berries) at harvest: C6-alcohol atoms

**Treatment**	**Exposed**			**Net-bagged**			**Boxed**		
	**μg/100 berries**								
	**mean**	**± SE**		**mean**	**± SE**		**mean**	**± SE**	
1- hexanol	26.05	0.29		26.5	0.35		32.95	2.84	n.s.
*Trans* 3-hexenol	1.45	0.04	a	0.9	0.06	b	1.55	0.02	a
Cis 3-hexenol	11.15	0.11	b	14.4	0.10	ab	17.95	1.58	a
*Trans* 2-hexenol	4.15	0.20	b	4.75	0.43	b	8.00	0.06	a

Values represent means ± SE (n = 3).

Lowercase letters indicate statistically significant differences at a 5% level of significance.

(Tukey’s HSD test); n.s. = not significant.

The terpenes released by enzymatic hydrolysis were detected in low quantities, mainly geraniol and its derivatives. In the boxed treatments, the amounts of geraniol, OH-geraniol and p-menth-1-ene-7,8-diol increased, while the quantity of *cis*-8-OH-linalool was lower than in the exposed treatments (Table 7). Total terpenes increased from 8.8% in the boxed treatment to 13% in the exposed treatment compared to the total aromas (Table 4).

**Table 7:** Glycosylated aromatic composition of grapes (μg/100 berries) at harvest: Terpenes

**Treatment**	**Exposed**			**Net-bagged**			**Boxed**		
	**μg/100 berries**								
	**mean**	**± SE**		**mean**	**± SE**		**mean**	**± SE**	
*Trans* furan linalool oxides	1.10	0.07		0.55	0.01		0.95	0.03	n.s.
*Cis* furan linalool oxides	0.45	0.03	b	0.50	0.01	b	1.70	0.05	a
Linalool	1.40	0.11		0.80	0.02		1.10	0.07	n.s.
α-terpineol	0.50	0.02	b	2.00	0.02	a	0.50	0.03	b
Geranial	0.95	0.05	b	0.75	0.04	b	1.70	0.04	a
*Trans* pyran linalool oxides	1.05	0.10	ab	0.70	0.16	b	1.60	0.17	a
*Cis* pyran linalool oxides	2.25	0.02	a	1.40	0.02	b	2.75	0.16	a
Citronellol	0.70	0.05	b	1.20	0.06	b	2.90	0.26	a
Nerol	3.65	0.18	b	3.10	0.43	b	6.60	0.23	a
Geraniol	13.65	0.55	b	13.50	0.06	b	24.50	2.41	a
2,6-dimethyl-3,7-octadien-2,6-diol	1.90	0.04	b	1.40	0.03	b	2.85	0.28	a
OH-citronellol	0.60	0.01	b	1.20	0.02	b	0.50	0.04	b
8-OH-dihydrolinalool	2.95	0.15	a	1.60	0.01	b	3.55	0.18	a
*Trans*-8-OH-linalool	7.35	0.34	a	5.20	0.17	b	8.35	0.51	a
*Cis-*8-OH-linalool	25.75	0.93	a	16.00	0.40	b	7.65	0.50	c
OH-geraniol	-	-		-	-		14.95	2.60	
Geranic acid	4.65	0.12		5.30	0.11		6.25	0.35	n.s.
*p*-ment-1-ene-7,8-diol	6.85	0.05	b	6.00	0.59	b	20.25	1.33	a

Values represent means ± SE (n = 3).

Lowercase letters indicate statistically significant differences at a 5% level of significance.

(Tukey’s HSD test); n.s. = not significant.

The C_13_-norisoprenoids were detected in low amounts compared to total aromas; they corresponded to 4.7% and 7.6% of the total aromas in the boxed and exposed clusters, respectively (Table 4). A 33.7% increase in the total C_13_-norisoprenoids was observed in the boxed treatments (Table 5), only 3-OH-β-damascone increased by 107% (Table 8).

**Table 8:** Glycosylated aromatic composition of grapes (μg/100 berries) at harvest: C13-Norisoprenoids

**Treatment**	**Exposed**			**Net-bagged**			**Boxed**		
	**μg/100 berries**								
	**mean**	**± SE**		**mean**	**± SE**		**mean**	**± SE**	
3-hydroxy-β-damascone	10.55	0.39	b	11.05	0.48	b	21.85	0.22	a
3-oxo-α-ionol	10.20	0.54	a	6.95	0.05	b	13.45	0.91	a
3.9-dihydroxy-megastigma-5-Ene	9.50	0.46	a	3.30	0.14	b	4.25	0.15	b
3-hydroxy-β-ionon	8.75	0.18	b	7.05	0.05	b	13.85	0.01	a
Vomifoliol	4.90	0.16	a	4.00	0.35	b	5.30	0.46	a

Values represent means ± SE (n = 3).

Lowercase letters indicate statistically significant differences at a 5% level of significance.

(Tukey’s HSD test); n.s. = not significant.

Total benzenoids were the most represented compounds (Table 4) and accounted for approximately 81.6% and 72% of the total aroma compounds in the boxed and exposed treatments, respectively. These compounds are responsible for vanilla, clove, almond, balsamic, resinous and moss flavours [40]. The boxed grapes had +141% of benzenoids than the exposed grapes (Table 5). However, benzyl alcohol and 2-phenylethanol, the main constituents, were detected in low quantities compared with the quantities found in other cultivars grown in Southern Italy [[41]. Benzyl alcohol demonstrated the most significant change in concentration: in the boxed berries, its concentration was 666.40 μg/100 berries; in net-bagged berries, its concentration was 253.60 μg/100 berries; in the exposed treatments, its concentration was only 173.25 μg/100 berries.

The concentration of 2-phenylethanol increased from 173.10 μg/100 berries in the exposed grapes to 207.10 μg/100 berries in the boxed treatments (Table 9).

**Table 9:** Glycosylated aromatic composition of grapes (μg/100 berries) at harvest: Benzenoids

**Treatment**	**Exposed**			**Net-bagged**			**Boxed**		
	**μg/100 berries**								
	**mean**	**± SE**		**mean**	**± SE**		**mean**	**± SE**	
Benzaldehyde	4.05	0.34	b	4.85	0.10	b	11.05	0.88	a
Methyl salicylate	4.70	0.28	b	5.80	0.08	b	15.35	1.26	a
Benzyl alcohol	173.25	4.46	b	253.60	22.54	b	666.40	36.37	a
2-Phenylethanol	137.10	0.40	b	195.75	2.58	a	207.10	17.62	a
Eugenol	5.10	0.16	b	8.40	0.17	b	23.35	1.55	a
Vanillin	1.30	0.06	b	1.90	0.01	b	3.45	0.21	a
Methyl vanillate	5.05	0.48	b	1.25	0.05	c	8.40	0.46	a
4-vinil guaiacol	1.25	0.09	b	1.65	0.04	b	3.70	0.37	a
Acetovanillone	12.80	1.07	b	13.40	1.27	b	26.60	0.31	a
Zingerone	2.80	0.15		1.65	0.01		2.85	0.31	n.s.
Homovanillic alcohol	64.75	1.78	a	31.75	0.27	b	26.00	1.04	b
Dihydroconiferyl alcohol	0.90	0.01		0.80	0.03		1.35	0.12	n.s.
Methoxyeugenol	0.55	0.04	b	0.60	0.02	b	1.85	0.13	a
Coniferaldehyde	4.00	0.12	b	1.60	0.11	b	8.85	0.22	a
Syringaldeide	-	-		0.50	0.05	b	2.15	0.15	a

Values represent means ± SE (n = 3).

## Discussion

Direct sun exposure greatly influenced bunch temperature. The boxed berries experienced the lowest levels of heat stress. The net-bagged berries had higher heat stress than the exposed berries, especially in the last two months (August and September).

The berry weight in the exposed treatment fluctuated during ripening due to the effects of direct sun that may have caused dehydration on hot days. The berries exposed to the net-bagged treatment were slightly lighter than the boxed grapes (which were the heaviest). Other authors using the same boxes did not report any differences in berry weight using Syrah [[14] or Pinot Noir grapes [[42]. The shading resulted in a delay in ripening, as previously reported with Cabernet Sauvignon [[3], Syrah [[22] and Nebbiolo grapes [[11]. Lower pH values and higher titratable acidity values (in particular, higher concentrations of malic acid) were found in fruit grown in the high light conditions. Sugar synthesis, which is due to the photosynthetic activity of leaves, should be independent of bunch shading [[9]. In other studies, using Syrah grapes [[14], using Pinot Noir grapes [[42] and using Merlot grapes [[43], there were no reported differences in the total soluble solids between shaded and exposed fruit when shading fruit with the same boxes. In our trials, the solid soluble content in boxed berries was always lower compared to other treatments [[4,6,7]. This finding may have been due to the delay in ripening, the presumable lower transpiration rate (lower bunch temperature) [44,45], and from the higher water content in boxed berries as it is supposed to the higher berry weight. The increase in the sugar levels in the net-bagged treatment in the last two sampling dates could be caused by a higher transpiration rate, due to the higher bunch temperatures observed in August, balanced by the phloematic inflow occurring in the last berry ripening stages [44,45].

Increased exposure to sunlight produced a decline in the titratable acidity due to malic acid degradation; this effect was enhanced by the high temperatures experienced by exposed fruit [[5]. Therefore, as expected, the boxed berries had the highest values of titratable acidity throughout the ripening season, followed by net-bagged berries and exposed berries.

Flavanols reactive to p-dimethylamino-cinnamaldehyde as (+)-catechin equivalents decreased with the decrease in light, and differences in the levels of flavanols were noted between the three treatments at harvest. Although net-bagged bunches experienced a decrease in incident radiation, there was sufficient light for the synthesis of proanthocyanidins; however, the complete absence of light significantly reduced the synthesis of these compounds [[2,21,42] due to the positive correlation between the concentration of skin tannins and sunlight incidence [[10,16]. In addition, the increasing heat in the exposed and net-bagged berries improved the proanthocyanidin content of grapes [[46].

The reduction in flavanols reactive to p-dimethylamino-cinnamaldehyde as (+)-catechin equivalents and total skin proanthocyanidins in boxed grapes represents an important finding for winemakers, as catechins are compounds that are easily released from the grape and can be oxidised by the polyphenoloxidase enzyme during the wine-making process, thus generating bitter and brown substances [27].

The potential flavour was characterised by a more abundant bound fraction compared to the free fraction, so the free terpenes in all treatments were absent. As previously determined in Muscat of Alexandria grapes [[24], the exposed and net-bagged grapes had similar concentrations of non-glycosylated and glycosylated aromatic components, and the levels in these grapes were lower than in the boxed fruits. In boxed berries, the temperatures may have been adequate for the biosynthesis of monoterpenes, but not high enough to cause excessive volatility of these compounds. Higher temperatures, such as those experienced in exposed and net-bagged berries, could have contributed to the enhancement of their biotransformation and degradation rate, while lower temperature values and light exposure in boxed grapes, increased the benzenoid concentrations [[47].

## Conclusion

The results of this trial elucidate that cv Grillo is characterized by a high reactivity to bunch microclimate condition.

Shade conditions in the bunch zone, due to high canopy density and/or different canopy management, would affect grape quality composition and therefore, the potential to made a wider range in wine styles.

## Competing interests

The authors declare that they have no competing interests.

## Authors’ contributions

PS conceived, designed and performed the experiment; analyzed the data, wrote the paper; AP conceived, designed and performed the experiment, analyzed the data, wrote the paper; DP carried out the chemical analysis and contributed to the data elaboration; PT was responsible for analysis of aromatic compounds; RDL conceived and designed the experiment; largely contributed to the manuscript revision; MGB conceived, designed and performed the experiment; analyzed the data, wrote the paper. All authors read and approved the manuscript.
